# Chimeric 2C10R4 anti-CD40 antibody therapy is critical for long-term survival of *GTKO.hCD46.hTBM* pig-to-primate cardiac xenograft

**DOI:** 10.1038/ncomms11138

**Published:** 2016-04-05

**Authors:** Muhammad M. Mohiuddin, Avneesh K. Singh, Philip C. Corcoran, Marvin L. Thomas III, Tannia Clark, Billeta G. Lewis, Robert F. Hoyt, Michael Eckhaus, Richard N. Pierson III, Aaron J. Belli, Eckhard Wolf, Nikolai Klymiuk, Carol Phelps, Keith A. Reimann, David Ayares, Keith A. Horvath

**Affiliations:** 1 Cardiothoracic Surgery Research Program, NHLBI, NIH, Bethesda, Maryland 20892, USA; 2 Division of Veterinary Resources, ORS, NIH, Bethesda, Maryland 20892, USA; 3 NICHD/NIH, Bethesda, Maryland 20892, USA; 4 Leidos Biomedical Research, Inc., Bethesda, Maryland 20892, USA; 5 University of Maryland Medical Center, Baltimore, Maryland 20201, USA; 6 MassBiologics, University of Massachusetts Medical School, Boston, Massachusetts 02126, USA; 7 Ludwig Maximilian University, Munich 81377, Germany; 8 Revivicor Inc., Blacksburg, Virginia 24060, USA

## Abstract

Preventing xenograft rejection is one of the greatest challenges of transplantation medicine. Here, we describe a reproducible, long-term survival of cardiac xenografts from alpha 1-3 galactosyltransferase gene knockout pigs, which express human complement regulatory protein CD46 and human thrombomodulin (*GTKO.hCD46.hTBM*), that were transplanted into baboons. Our immunomodulatory drug regimen includes induction with anti-thymocyte globulin and αCD20 antibody, followed by maintenance with mycophenolate mofetil and an intensively dosed αCD40 (2C10R4) antibody. Median (298 days) and longest (945 days) graft survival in five consecutive recipients using this regimen is significantly prolonged over our recently established survival benchmarks (180 and 500 days, respectively). Remarkably, the reduction of αCD40 antibody dose on day 100 or after 1 year resulted in recrudescence of anti-pig antibody and graft failure. In conclusion, genetic modifications (*GTKO.hCD46.hTBM*) combined with the treatment regimen tested here consistently prevent humoral rejection and systemic coagulation pathway dysregulation, sustaining long-term cardiac xenograft survival beyond 900 days.

## Abstract (web summary)

Tweaking immune characteristics of donors and recipients could allow for successful cross-species organ transplantation. Here, the authors show that an anti-CD40 antibody therapy of baboons that received heart transplants from genetically modified pigs is key to their long-term survival.

## Graphical abstract

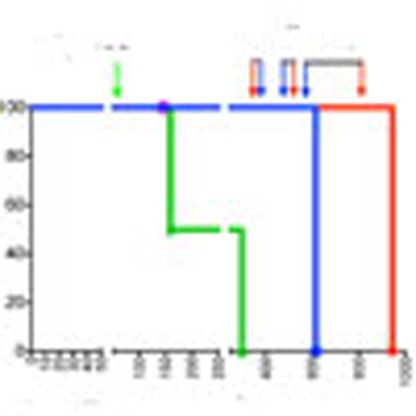

Previous pig-to-primate xenotransplantation studies have been beset with treatment-related complications and delayed xenograft rejection characterized by thrombotic microangiopathy (TM) and consumptive coagulopathy (CC)^1^,^2^,^3^. Previously, the longest survival for heterotopic cardiac xenografts using *alpha 1-3 galactosyltransferase* gene knockout (*GTKO*) or *GTKO.hCD46* Tg pigs and immunosuppression (IS) that included αCD154 antibody was 179–236 days, however, the xenografts ultimately succumbed to the characteristic properties of delayed rejection^4^,^5^. We have recently reported survival beyond 1 year of *GTKO.hCD46*.*hTBM* pig heart xenografts that were heterotopically transplanted into a baboon. The immunomodulatory protocol utilized αCD40 antibody, documenting an attenuated anti-pig antibody elaboration, absence of TM in the graft and reduced incidence of systemic CC^6^. Longer-term and reliable prevention of xenograft rejection in this preclinical model has not previously been described.

In the present study, using baboon recipients of *GTKO.hCD46.hTBM* porcine hearts, we evaluated an iterative modification of our previous immunomodulation strategy, one that primarily targets the anti-CD154-CD40 co-stimulation pathway. We report consistent prevention of xenograft rejection in association with ongoing αCD40 (2C10R4) antibody treatment resulting in graft survival beyond 2 years.

## Results

### Anti-CD40 significantly prolongs graft survival

**Figure 1:**
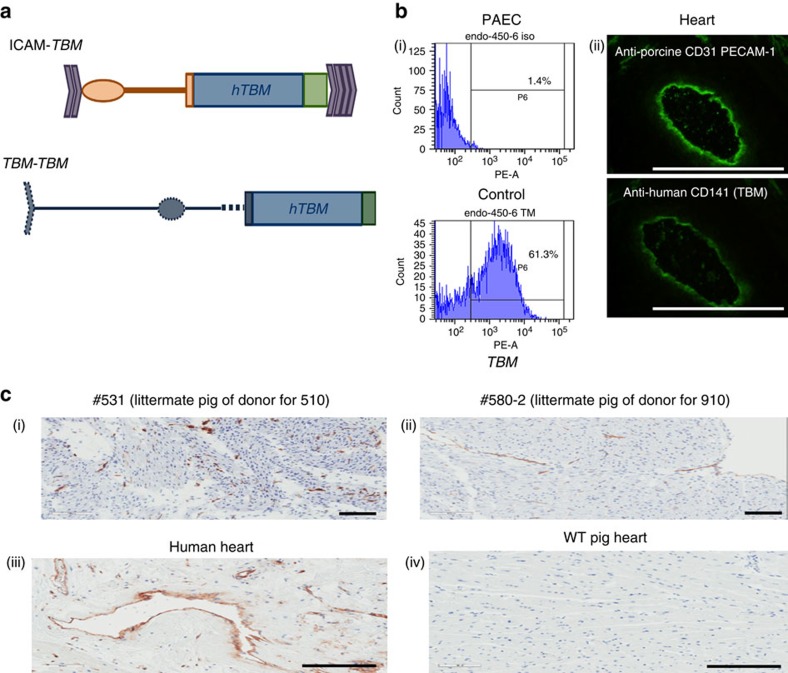
Expression of *TBM* transgene. (**a**) Donor pigs with two distinct *hTBM* transgenes were used. ICAM-*TBM* and *TBM*-*TBM*. (**b**) Describes the *hTBM* expression in porcine aortic endothelial cell (PAECs) from donor pig using FACS analyses (i) and immunohistochemistry staining (ii) of pig heart tissue using human anti-porcine CD31 antibody. (**c**) Demonstrates the TBM molecule expression in heart tissue from the littermates of donor pigs for baboons #910 and #510 (i and ii), human heart section (iii) and wild-type pig heart section (iv). Scale bar, 50 μm.

The rationale for using *GTKO*) pigs to avoid anti-Gal antibody-mediated rejection, with additional human complement regulatory protein (hCD46) expression to suppress complement activation, has been previously reported^5^. Similarly, the benefits of anti-CD154/CD40, αCD20 and mycophenolate mofetil (MMF) immunosuppressive treatment on xenograft survival prolongation in baboons have been described^4^,^5^,^6^,^7^,^8^. We used our established heterotopic cardiac xenotransplantation model using *GTKO.hCD46* porcine donors^9^,^10^, engineered to additionally express the human thromboregulatory protein, human thrombomodulin (*hTBM*)^11^,^12^. This genetic modification is intended to reduce thrombo-dysregulation often associated with xenografts. Specific pathogen-free baboons received a co-stimulation pathway-blocking drug regimen described in Table 1, including a non-depleting primatized αCD40 monoclonal antibody (2C10R4) (ref. 13). Postoperatively, a telemetry implant was used to continuously monitor graft contractility, electrocardiogram (EKG) and body temperature^14^. Telemetric surveillance was supplemented with constant video monitoring of the recipient baboon. Hearts from *GTKO.hCD46* pigs engineered using one of two *hTBM* gene constructs with different promoters (*ICAM* in baboons #110, #210 and #910 and *TBM* in baboons #15009 and #510) were used (Fig. 1a; cloned from founder lines selected for high endothelial expression of *hTBM*). This was the first time this *hTBM* transgene was used and the efficacy of the two available promoters was not known. The *ICAM-2* promoter was clearly superior in expression/functionality of *TBM* in endothelial cells, both by flow cytometry and generation of antigen presenting cell (APC), when compared with endothelial cells from the *pTBM-TBM* pigs. However, immunochemistry of heart tissue (*n*=1 for each promoter type) indicated more robust expression of *hTBM* generated by the porcine endogenous *TBM* promoter. Both promoters provided significant expression of *TBM* on the cardiac vascular endothelium. Overall, the transplanted *TBM*^+^ hearts with the *TBM* promoter perform as well or better than the ones bearing the *ICAM-2* promoter. Histologic depiction of the representative expression of *hTBM* on porcine aortic endothelial cells (PAECs; Fig. 1b) and on the endothelial cells of donor pig littermates (Fig. 1c(i,ii)) is illustrated relative to human vascular endothelial cells (Fig. 1c(iii)). As expected, *hTBM* expression is not detected in the wild-type pig (Fig. 1c(iv)).

**Figure 2:**
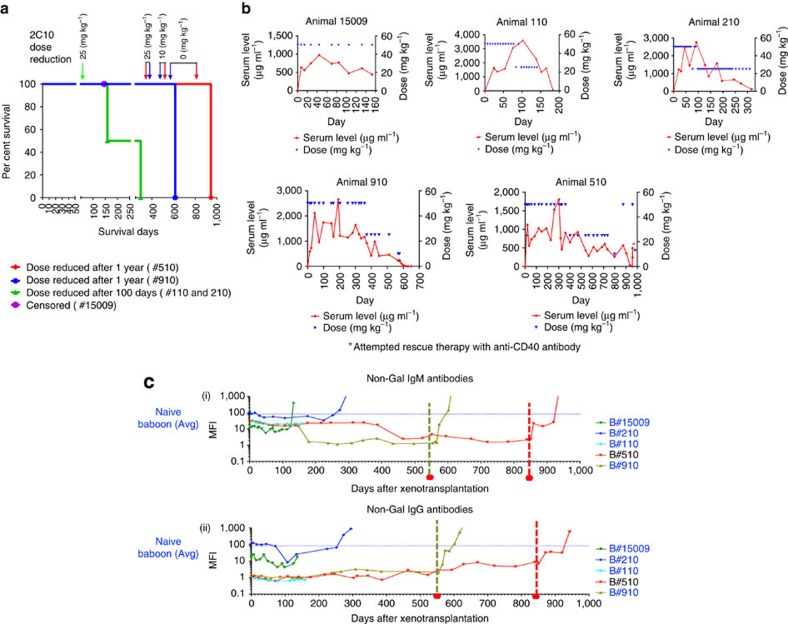
Cardiac xenograft survival. (**a**) Survival graph showing cardiac xenograft survival for five baboons. It also indicates the time points when the 2C10R4 dose was reduced. The long-term graft survival in two animals is plotted separately to give clear idea of the time points for antibody dose reduction. In two animals, the dose was reduced to 25 mg kg^−1^ on day 100 (green arrow); in two long-term surviving animals dose reduction to 25 mg kg^−1^ was made after 1 year and the antibody treatment was terminated in #910 on day 560 (blue arrow) and in #510 on day 861 (red arrow). (**b**) Detection of 2C10R4 levels in the plasma of the transplanted baboons. A blue triangle represents each dose. (**c**) Non-Gal (both IgM and IgG) antibody production in all baboons. The green and red dashed lines indicate the day αCD40 antibody treatment was stopped for baboons #910 and 510, respectively.

As demonstrated in Fig. 2a, all five *GTKO.hCD46.hTBM* hearts had significantly prolonged xenograft survival (mean 433 days, median 298 days and range 159–945 days) compared to previously reported *GTKO.hCD46* pig heart survival using αCD40 antibody at a lower dose (longest survival 146 days)^7^ and the same group of animals, described in this study, at an earlier stage of survival (longest survival >500 days)^6^. One animal that succumbed to an antibiotic-resistant infection with a contracting graft at 146 days was censored from the survival analysis; a source of infection was not established on necropsy.

When αCD40 antibody was administered at a lower dose (25 mg kg^−1^) and tapered off over the course of 2 months, none of the *GTKO.hCD46*.*hTBM* grafts survived long term^7^. In contrast, significant survival was achieved when a higher dose of antibody (50 mg kg^−1^) was used^6^. We then elected to decrease the weekly dose of weekly αCD40 antibody in two animals to 25 mg kg^−1^ after 100 days. Both grafts slowly failed in association with recrudescence of anti-donor antibody. In contrast, the two recipients receiving a high dose of antibody maintained cardiac function despite gradual reduction in the antibody dose after 1 year, as illustrated in Fig. 2. The αCD40 antibody was finally discontinued in baboon #910 on day 560 and in baboon #510 on day 861. Both grafts rejected after 8–10 weeks of antibody termination on days 616 and 945, respectively. Progressive graft dysfunction occurred concomitant with the washout of αCD40 antibody (Fig. 2b) and subsequent return of anti-pig antibody (Fig. 2c).

### Laboratory and mechanistic correlates

**Figure 3:**
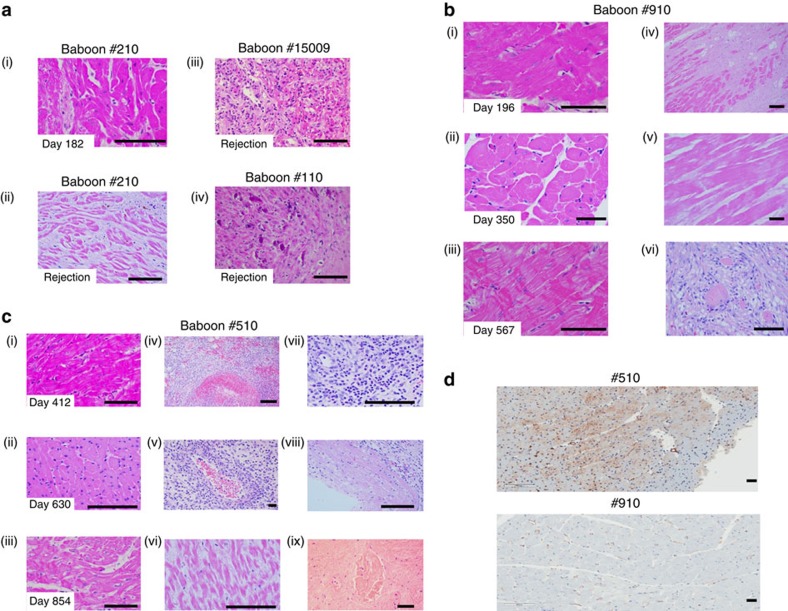
Histology of cardiac xenografts. (**a**) Graft biopsy on day 182 (i) in baboon #210, demonstrating normal myocardial architecture; terminal histology of grafts from baboons #210 (ii, illustrating myocyte necrosis; #15009 (iii, showing focal myocarditis; and #110 (iv, showing myofibril loss with mineralization; × 400). (**b**) Baboon #910. Left panel shows normal histology of biopsy specimens obtained on days 196 (i), 350 (ii) and 567 (iii); right panel illustrates terminal histology, with multifocal fibrosis (iv), coagulative necrosis (v) and venular thrombi (vi). (**c**) Baboon #510. Left panel shows normal histology from biopsies on days 412 (i), 630 (ii) and 854 (iii). Middle and right panels show terminal graft histology, illustrating haemorrhage (iv), arteritis (v), coagulative necrosis (vi), neutrophilic infiltrates (vii), epicardial infiltrates (viii) and venular thrombi (ix). (**d**) Thrombomodulin expression in explanted grafts from baboons #510 and #910 by immunohistochemistry (IHC). Scale bar, 50 μm.

Histology of the grafts in baboons that rejected after αCD40 dose reduction on day 100 demonstrated features typical of xenograft rejection, including myocyte death, haemorrhage, fibrosis, with sparse cellular infiltration by macrophages, neutrophils and occasional lymphocytes (Fig. 3a(ii,iv)). The histology of the graft from baboon #15009 that succumbed to infection with a contracting graft on continued αCD40 antibody treatment demonstrated focal myocarditis (Fig. 3a(iii)). There was no evidence of TM in the graft or CC in any baboon during ‘full-dose' antibody treatment (Fig. 3a(i)). After terminating the αCD40 antibody treatment, long-term surviving hearts demonstrated a typical xenograft rejection pattern, including characteristic features of TM, vasculitis, intravascular thrombus, myocardial necrosis and epicardial haemorrhage. There was little or no interstitial lymphocytic infiltration identified in these grafts (Fig. 3b,c). The biopsies from both long-term animals demonstrated sustained levels of *hTBM* expression (Fig. 3d) similar to untransplanted hearts from littermates (Fig. 1c(i,ii)).

**Figure 4:**
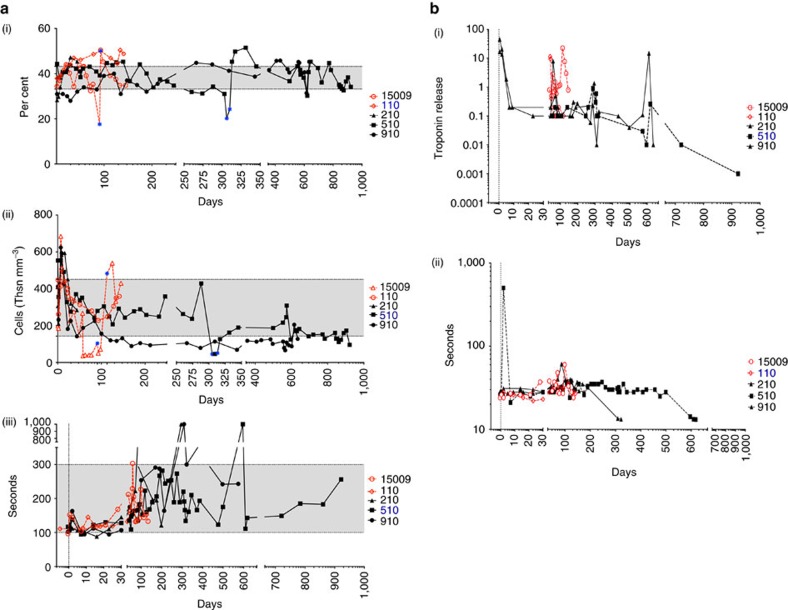
Laboratory blood test. (**a**) Illustrates the levels of haematocrit (i), platelets (ii) and activated clotting time (iii) in each experimental animal. (**b**) Demonstrates the levels of troponin (i) and prothrombin time until day 600 (ii).

**Figure 5:**
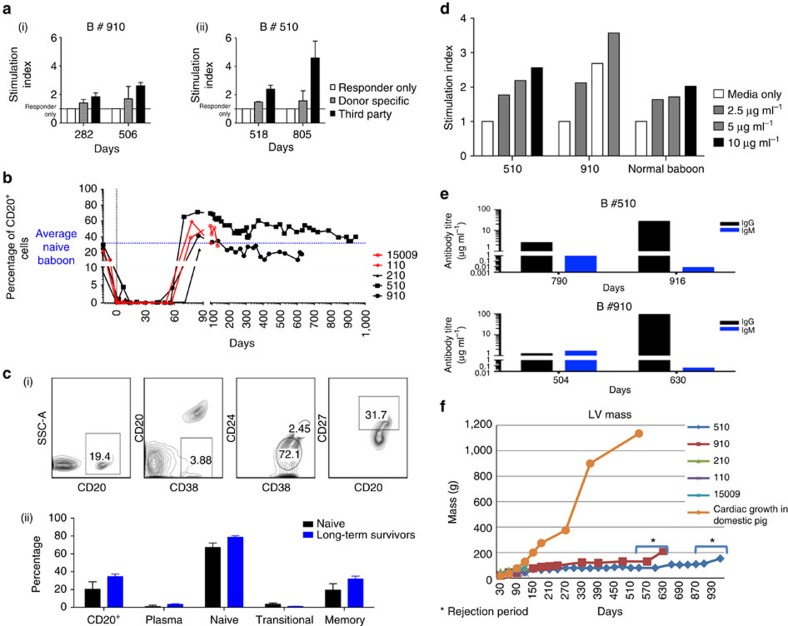
*In vitro* cellular assays. (**a**) Mixed lymphocyte reaction of cells from baboons #910 (i) and #510 (ii). Two control specimens were used for each assay and each specimen was plated in triplicates; s.d. is also shown with bars. (**b**) Suppression of B-cell numbers in all five baboons for 60 days after αCD20 antibody treatment. (**c**)—(i) Describes the phenotypes of B lymphocytes from an untreated baboon; (ii) comparison of an untreated and experimental baboon (#510) phenotype with s.d. (**d**) Proliferation of B lymphocytes of naive, baboons #510 and #910 in response to human IgM antibody stimulation at one time point. (**e**) Antibody production from human IgM-stimulated B cells from long-term surviving baboons #510 and #910 at one time point. (**f**) The line graph demonstrates the rate of change in graft size in all five baboons. The time from termination of the 2C10R4 treatment to rejection is also indicated.

As shown in Fig. 4a(i), the haematocrit (HCT) data for all five baboons indicate that a normal complete blood count profile was maintained during treatment. Distinct from our work using *GTKO.hCD46* hearts and CD154-based IS^5^, platelet numbers were maintained within the normal range (Fig. 4a(ii)), and no spontaneous bleeding episodes were observed despite continuous heparin administration (activated clotting time (ACT) goal=2 × baseline level; Fig.4a(iii)). These observations clinically demonstrate an absence of CC, which is a hallmark of delayed xenograft rejection. Both prothrombin time and troponin^15^,^16^, remained within normal limits or undetectable in all baboons until IS reduction and subsequent onset of rejection (Fig. 4b(i,ii)). Representative mixed lymphocyte reaction (MLR) data (Fig. 5a) illustrate donor-specific suppression of T-cell proliferation in baboons #510 and #910 one year after transplantation while receiving αCD40 treatment, with preserved proliferative response to third party pig antigens.

Following induction treatment with αCD20 antibody after transplantation, B cells in the recipient baboons were not detected in peripheral blood for over 60 days (Fig. 5b). During subsequent recovery, the circulating B-cell subsets were phenotypically similar to the normal baboon B-cell subsets (Fig. 5c(i,ii)). The B lymphocytes in peripheral blood lymphocytes proliferated well in response to anti-IgM antibody stimulation (Fig. 5d), but antibody elaboration remained suppressed *in vitro* while αCD40 antibody remained detectable in the recipient's serum (Fig. 5e).

Analysis of physiological aspects of pig-to-primate xenotransplantation suggest that the transplanted pig organ is responsive to primate growth hormone, and thus can grow to a size proportional to the primate's organs^17^,^18^. As shown in Fig. 5f, left ventricular mass, estimated based on serial echocardiograms, increased three to fivefold in size over the first year. However, in hearts surviving for over 1 year, this growth plateaued at a mass ∼60% that of a normal adult human heart. Both the transplanted pig heart and the recipient baboon heart were of the same size at this stage. After ceasing αCD40 antibody treatment, further preterminal graft enlargement was observed, histologically proven to be myocardial oedema and haemorrhage. Before preterminal rejection, growth of heart xenografts remained <30% that of hearts in age-matched domestic pigs^19^. This indicates that the organs did not exhibit overgrowth to a size too large for the recipient.

## Discussion

Until recently, all attempts using conventional or experimental immunosuppressive regimens and various genetically engineered donor pigs have yielded inconsistent short-term success, with substantial recipient morbidity and graft survival typically <9 months^4^,^5^,^7^. Although further improvements may be necessary for both donor genetics and recipient treatment to achieve consistent orthotopic heart xenograft performance and justify potential clinical applications, our data show that the use of *GTKO.hCD46.hTBM* donor pigs and an αCD40 (2C10R4) antibody-based immunomodulatory regimen is associated with significantly prolonged graft survival. This regimen was well tolerated in baboons with no evidence of significant infections related to IS. In our opinion, this regimen appears potentially safe for human application for patients suffering from end-stage organ failure who might be candidates for initial trials of xenotransplantation. Our future efforts are to improve this immunomodulatory regimen to a level that is analogous to what is used for allotransplantation and to further tailor the immunosuppressive regimen accordingly when a less immunogenic ideal genetically engineered donor is available.

Although the expression of human complement pathway regulatory proteins^8^,^20^,^21^, the absence of Gal epitope in *GTKO* pigs and both modifications together^5^,^22^,^23^ reduced the incidence of hyperacute rejection and early graft failure, these modifications have proven insufficient to prevent the delayed antibody-associated xenograft rejection in the context of intensive conventional or experimental IS. This form of rejection is characterized by TM in the graft and CC in the recipient^4^,^24^,^25^,^26^. Recently, the role of a carbohydrate antigen *N*-glycolylneuraminic acid (Neu5GC) in xenograft rejection in humans has been described^27^. Baboons, however, do not have antibodies against this carbohydrate as they naturally express Neu5GC antigen, and therefore the impact of Neu5GC on graft rejection cannot be studied in this model^28^. Recently, pigs have been engineered where *CMP-N-acetylneuraminic acid hydroxylase (CMAH)*, the gene for the enzyme that is responsible for depositing this carbohydrate on the endothelial surface, has been knocked out. Removal of this carbohydrate antigen from the pigs will likely eliminate rejection due to anti-Neu5GC antibody. In addition, based on known interspecies molecular incompatibilities^29^ and coupled with direct evidence that organ and systemic coagulation pathway dysregulation is implicated in late xenograft rejection, several human thromboregulatory molecules are being explored to inhibit primary (due to intrinsic interspecies molecular incompatibilities) or elicited (by innate or adaptive immune injury) mechanisms suspected of contributing to the TM and CC phenotypes.

For *GTKO* organs, with or without additional genetic modifications, xenograft rejection is generally associated with a robust antibody response directed against multiple non-Gal carbohydrate and protein antigens^30^. Although anti-non-Gal antibody is not always detected at the time of graft or recipient demise, we hypothesized that incomplete control of elicited anti-pig immunity associated with previous regimens constitutes a key barrier to clinical application.

Historically, IS using ‘conventional', clinically used agents have been relatively ineffective to prevent xenograft injury and associated phenomena, even at doses associated with significant recipient morbidity and mortality^3^,^31^. Depletion of B cells using αCD20 antibody was associated with improved xenograft and recipient survival when administered with conventional IS^3^ or co-stimulation pathway blocking agents. On the basis of our experience using blockade of the CD40/154 pathway combined with of induction anti-thymocyte globulin (ATG), αCD20 antibody^5^,^7^,^8^,^23^ and ongoing MMF, the primary aim of this study was to evaluate an intensified immunomodulatory αCD40 regimen in concert with *GTKO.hCD46*.*hTBM* grafts.

Here we report xenograft survival consistently extending beyond 4 months (median of 298 days) in five consecutive recipients of heterotopic *GTKO.hCD46.hTBM* heart xenografts treated with a clinically applicable, intensified αCD40 antibody maintenance regimen, and graft survival prolongation with normal histology beyond 1 year in two cases. Although immunologic tolerance was not achieved, donor-specific hypo-responsiveness was observed, and immunoregulation was maintained in the T- and B-cell compartments during ongoing αCD40 antibody and MMF treatments in two long-term animals. Remarkably, CC and TM were not observed during treatment.

We noted that B cells failed to produce antibodies despite the reconstitution of naive B-cell phenotypic profile in peripheral blood 2–4 months after cessation of αCD20 antibody treatment. Although induction ATG and αCD20 antibody and ongoing MMF therapy may contribute to this, we speculate that sustained suppression of B-cell function is primarily attributed to the ongoing αCD40 antibody treatment since IgM-stimulated B-cell antibody production *in vitro* and elaboration of anti-pig antibody was observed *in vivo* around the time αCD40 antibody became undetectable in the serum, and in the context of ongoing MMF treatment. Indeed, we consider it likely that both αCD40 antibody (by mediating control of anti-pig B-cell maturation) as well as the *hTBM* transgene (by reducing the consequences associated with one of several identified interspecies coagulation pathway incompatibilities) were necessary to achieve the observed xenograft survivals. As in previous work, removal of αCD40 antibody resulted in graft loss, underscoring the importance of this pathway to regulate xenograft rejection or acceptance. An important role for *hTBM* is suggested by our current work, as we have shown that ongoing treatment with the anti-CD154-CD40 co-stimulation blockade antibody is associated with inconsistent protection of *GTKO.hCD46* grafts from TM with graft survival limited to 8 months. It is mechanistically plausible that interplay between coagulation, anti-pig antibody, complement and T cells contributed to xenograft rejection. Furthermore, one important observation was the absence of CC, a hallmark of delayed xenograft rejection^1^,^2^,^3^,^8^ in all five animals. It is likely that the three genetic modifications used here (*GTKO.hCD46.hTBM*) played a significant role in avoiding this complication.

Although additional work will be necessary to determine which components of this pharmacologic regimen are necessary, in addition to optimizing drug dosing and evaluating the importance of the *hTBM* transgene, this result represents an important finding for the field of xenotransplantation. Its potential clinical relevance is underscored, since several non-depleting αCD40 antibodies are in late-stage preclinical development (clinicaltrial.gov identifier NCT01561911, NCT00001789).

In addition, there was a concern that the pig heart, with its normal development, may grow out of the abdominal cavity. Our serial echocardiographic estimates of cardiac xenograft volume throughout a 2-year time span suggest that juvenile pig grafts exhibit neither supraphysiologic growth nor pathologic hypertrophy in this heterotopic transplantation model.

In conclusion, the current results confirm the pivotal importance of the CD40-CD154 co-stimulation pathway in the immunopathogenesis of xenograft rejection. Furthermore, they support our working hypothesis that local expression of human thromboregulatory molecules by a *GTKO.hCD46* graft is likely sufficient, and almost certainly necessary to prevent coagulation-related xenograft injury and associated pathology in the context of the regimen employed here. Further work is required to confirm these current findings and test them in a life-supporting preclinical model. If successful, the approaches described here could help translate xenotransplantation of the heart and other organs into a potentially transformative therapeutic option for the thousands of transplant candidates who may benefit from the timely availability of a porcine organ.

## Methods

### Animals

Only specific pathogen-free baboons (*Papio anubis*) of either sex weighing 7–15 kg from University of Oklahoma (Norman, OK) were housed in a clean pathogen-free facility and were used as recipients of 2–3 years of age. Six- to eight-week-old genetically modified *GTKO* pigs of either sex with an overexpression of human *CD46* and *TBM*, that is, *GTKO.hCD46.*hTBM** pig (Revivicor Inc., Blacksburg, VA) were used as donors. Two different promoters were used for the expression of *TBM*. The *ICAM* promoter was used for the donor pig hearts transplanted in baboons #110, #210 and #910 and the *TBM* promoter was transplanted in baboons #510 and #15009. The expression of transgene was stable at the genomic level, and expression of hCD46 and TBM protein is consistent and high level across all *GTKO.CD46.TBM* pigs. During selection of donor pigs special consideration was given to the approximate size of the hearts and their ability to fit in baboon's abdomen. All animals were used in compliance with guidelines provided by the National Heart, Lung and Blood Institute (NHLBI) Animal Care and Use Committee (ACUC).

### Immunosuppression

Immunosuppressive regimen (Table 1) for all recipient baboons included induction therapy which comprised ATG (Thymoglobulin; Genzyme, Cambridge, MA, USA); 5 mg kg^−1^ on days −2 and −1), αCD20 antibody (Rituxan; Genetech, San Francisco, CA, USA; 19 mg kg^−1^ on days −14, −7, 0 and 7) for T- and B-cell suppression and αCD40 (clone 2C10R4)^15^ (NHP Reagent Resource, Boston, MA, USA; 50 mg kg^−1^ on days −1, 0, 5, 9, 14 and then 1 week) for blocking the co-stimulation pathway. Cobra venom factor (Quidel, San Diego, CA, USA; 1,500 units; −1, 0 and 1) was used to inhibit the complement activation. To maintain the long-term IS, MMF (Genzyme; 20 mg kg^−1^ BID) was used daily and αCD40 antibody (50 mg kg^−1^) was infused weekly for 100 days (*n*=2) or for 1 year (*n*=2). After 1 year, the antibody dose was first reduced to 25 mg kg^−1^ and then was gradually tapered off. All recipient baboons received continuous heparin infusion to keep the ACT level twice the baseline. Ganciclovir (Roche, Nutley, NJ, USA; 5 mg kg^−1^ per day) was administered daily to prevent the viral infections. Epogen (Amgen, Thousand Oaks, CA; 200 U kg^−1^) was administered daily from day −7 to 7 to maintain the HCT and Cefazolin (Hospira, Lake Forest, IL, USA; 250 mg) was given twice a day for 7 days to prevent infections.

At the earliest sign of rejection, demonstrated by slowing down of graft contractility, rescue therapy was initiated with intravenous bolus dose of methylprednisolone (125 mg) and then was continued (10–15 mg kg^−1^) for next 6 days. Increased heparin dosage was also used to prevent thrombus formation and ACT was maintained twice the baseline by using heparin. A full dose (50 mg kg^−1^) of αCD40 antibody was used to rescue graft of baboon #510 at the time of rejection.

### Heterotopic transplantation procedure of the xenograft

All transplant procedures were performed at an NHLBI core surgical facility.

Donor pig's chest is opened by a midline incision extending from xiphoid to the manubrium. Both inferior and right superior vena cava are ligated to occlude venous return, and the inferior vena cava, left superior vena cava and left atrium are incised. A cardioplegia catheter in the ascending aorta delivers cold University of Wisconsin solution proximal to an aortic cross-clamp as crushed saline ice is applied in the pericardium. The heart is removed by dividing the cavae, left atrium, pulmonary artery and aorta, leaving a long enough great vessels for the anastomosis. The left atrium and cavae are oversewn after the heart is taken out of the chest cavity.

The recipient baboon's abdomen is opened by a midline incision and infrarenal abdominal aorta and inferior vena cava are exposed and isolated. After heparinization (dose), side-biting clamps are applied, and aortotomy and venotomy are made. Donor aorta is anastomosed end-to-side with the recipient's aorta, and donor pulmonary artery to the recipient's inferior vena cava. After clamp removal, graft decompression is accomplished manually until return of cardiac function and restoration of sinus rhythm.

### Methods for evaluating xenograft functions

Xenograft functions were evaluated by telemetry (continuously; 24/7 hours per day a week), palpation and ultrasound. Telemetry device (Konigsberg Instruments, Inc., Pasadena, CA, USA) was implanted into the recipient baboon to monitor the xenograft left ventricular pressure (LVP), ECG, and recipient's temperature. The telemetry device data were transmitted wirelessly to a receiver (RMISS, Wilmington, DE, USA) attached to the animal's cage, which has also recorded peak systolic pressure (PEAK), end diastolic pressure (END), LVP (PEAK-END), heart rate based on LVP (LVPHR), EKG, heart rate based on EKG (EKGHR) and recipient's body temperature (TEMP). LVP >60 mm Hg was considered normal cardiac xenograft function. Any drop in LVP below 60 mm Hg was considered the point at which the rejection process started to affect the graft contractility. And if, LVP falls below 10 mm Hg, it was an indication of complete cessation of xenograft contractility. Palpation of xenograft was recorded (++++ for fully functional to 0 for rejection) and/or ultrasound was used to confirm the graft status. Final diagnosis of rejection was made by histopathology.

### Ultrasonography of cardiac xenograft

A two-dimensional echo was performed on five baboons after xenotransplantation surgery and followed at a minimum monthly to estimate LV mass during the study period (0–945 days). The LV mass was calculated based on a cross-sectional area *x* length formula using the observed myocardial cross-sectional area at the mid-papillary muscle on a short-axis view during end diastole. Image selection was based on the ability to clearly identify cardiac structures (endocardium, epicardium, apex and mitral value). For each echo, measurements were repeated five times with the average value being used within the calculations. Calculated LV mass were averaged within 30-day time periods for the duration of the study period and graphed using excel.

### Haematological and biochemical parameter of recipients

Complete blood count, which includes white blood cell counts, hematocrit, red blood cells, haemoglobin, platelets, neutrophils and monocytes were analysed by HemaVet 950 hemoanalyser and histochemistry was performed weekly for the first 2 months and then biweekly until the xenograft is explanted. ACT and troponin levels were measured by iStat (Abbott Laboratories, Princeton, NJ, USA).

### Measurement of non-Gal IgG and IgM antibodies

PAECs were isolated and cultured from *GTKO* or *GTKO/CD46T*g pigs. Non-Gal antibodies (IgG and IgM) titre was measured in serum by flow cytometry. Serum samples collected from baboon before and after transplant every 2–3 days for month and biweekly thereafter. Flow cytometry was used to measure antibody binding (mean fluorescence intensity) with FITC-labelled anti-human IgG (Cat# H10301) and IgM (Cat# H15101) antibodies (Invitrogen Corp., Waltham, MA, USA) to PAECs on a LSRII (Becton Dickinson, San Francisco, CA, USA) cytometer. The mean fluorescence intensity of the cells were analysed with FlowJo software (FlowJo LLC, Ashland, OR, USA) for each test serum and compared with that produced by the controls.

### Detection of αCD40 antibody in baboon plasma

Detection of recombinant αCD40 in the plasma was assessed by enzyme-linked immunosorbent assay (ELISA). Plates were coated with recombinantly expressed extracellular domain of rhesus CD40 protein fused to maltose binding protein (NHP Reagent Resource) at a concentration of 0.01 mg ml^−1^ and blocked with Super Block (Thermo Scientific, Woodstock, GA, USA). Pre- and post-treatment samples were serially diluted, plated for 1 h, and washed with PBS/0.05% Tween 20. Circulating αCD40 antibody, 2C10R4, was detected by incubating with polyclonal donkey anti-human IgG (H+L)—horseradish peroxidase (Jackson ImmunoResearch Labs, West Grove, PA, USA). Plates were then incubated with 1-Step Ultra TMB ELISA Substrate (Thermo Scientific). TMB stop solution (Cat# 52-00-01; KPL, Gaithersburg, MD, USA) was added and the absorbance was read on Mithras LB 940 microplate reader (Berthold Technologies, Calmbacher, Germany) at 450 nm. A standard curve was generated with serum spiked with known quantities of αCD40 and was used to calculate the αCD40 concentration present in the serum.

### Measurement of αCD40 antibody levels

Baboon antibody response to αCD40 was measured by ELISA. Ninety-six-well plates were coated overnight with treatment antibody at 0.01 mg ml^−1^ in PBS and then blocked with Super Block (Thermo Scientific) for 15 min. Pre- and post-treatment plasma samples obtained from each monkey were serially diluted in PBS/2% FBS and applied to the plates for 1 h. Plates were then washed 10 times with PBS/0.05% Tween and incubated for 1 h. with biotinylated goat anti-human lambda chain (Miltenyi Biotec Inc., San Diego, CA, USA). This antibody cross-reacts with baboon lambda light chain but does not recognize the treatment antibody, which has kappa light chains. Plates were then incubated with 1-Step Ultra TMB ELISA Substrate (Thermo Scientific). TMB stop solution (KPL) was then added to each plate and OD read on Mithras LB 940 microplate reader (Berthold Technologies) at 450 nm. A sample was considered positive at a given dilution if the OD reading of the post-treatment plasma exceeded the OD of the pre-treatment at the same dilution by twofold.

### T- and B-cell phenotyping by FACS analysis

Immuno-staining was performed on peripheral blood mononuclear cells (PBMCs) with fluorescence-conjugated antibodies. Anti-human CD3 (Cat# 556112), CD4 (Cat# 560811), CD20 (Cat# 560736), CD27 (Cat# 557329), CD38 (Cat# 561106), IgD (Cat# 563313) and IgM (Cat# 562618) monoclonal antibodies from Pharmingen (BD Bioscience, San Francisco CA, USA) were used. Anti-CD19 (Cat# 3602211), CD24 (Cat# 311120) from BioLegend, San Diego, CA, USA and anti-CD5 (Cat# MHCD0518) from Invitrogen Corp. was used. Antibodies (3 or 4 μl per million cells) were used as recommended or suggested by the manufacturers. Samples were run on FACS Calibur or LSR2 (BD Bioscience). Flow cytometry analysis was performed using FlowJo software (FlowJo LLC). The samples from long-term survivors were analysed and s.d. was calculated and plotted along with the data.

### Immunological assessment of host immune response

PBMCs from recipient baboon and donor pigs were isolated using Ficoll-Hypaque gradient method^12^ for MLR. Donor PAECs were isolated as follows: segments of donor porcine pulmonary artery were obtained and rinsed in sterile DMEM (pH 7.4; Gibco, Grand Island, NY, USA), containing 200 U ml^−1^ penicillin, 200 μg ml^−1^ streptomycin, and 325 μg ml^−1^ fungizone and then washed twice with PBS. The artery was then trimmed for any extra tissue and then soaked in 0.25% trypsin-ETDA solution in DMEM at 37 °C for 20–30 min. Cell suspension was centrifuged at 1,000*g* for 10 min at 4 °C. The supernatant was discarded and the pellet was washed two times with cold medium. T-25 flasks were seeded with 1.5–3 × 10^5^ and capped loosely and placed in a CO_2_ incubator at 37 °C (5% CO_2_ until cells becomes confluent). The medium was changed every 2 days. Both donor PBMCs and PAECs were kept frozen for future use as stimulators or antigen-presenting cells in MLR. Baboon PBMCs (5 × 10^4^) were co-cultured with irradiated donor pig PBMCs (5 × 10^4^) or PAECs (5 × 10^3^) in triplicate in 96-well round-bottom tissue culture plates (Cat# 3799; Corning Costar, Corning NY, USA) with a final volume of 200 μl complete RPMI (Cat# 22400-097; Invitrogen Corp.; cRPMI: RPMI, 10% FCS, 50 mg ml^−1^ Gentamicin, 2 mM L-glutamine). The cells were cultured for 5 days in a humidified incubator with 5% CO_2_. Proliferation was assessed by the MTT-based CellTiter 96 AQueous One Solution Cell Proliferation (Cat# G3580; Promega Corporation, Madison, WI, USA) kit. MTS tetrazolium compound is bioreduced by cells into a coloured formazan product that is soluble in tissue culture medium. The quantity of formazan product measured by the absorbance at 490 nm is directly proportional to the number of living cells in culture. The stimulation index (SI) was calculated by dividing the MLR (contains responder cells* and non-proliferating stimulator cells**) by the non-stimulated responder cell* response, where responder cells were from baboon recipient and stimulator cells were irradiated pig PBMCs or PAECs.


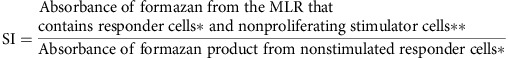


*Responder cells: baboon PBMCs; **non-proliferating stimulator cells: irradiated donor pig PBMCs or PAECs.

Isolated baboon recipient PBMCs were also stimulated with 10 μg ml^−1^ of anti-IgM (Fab_2_) antibody (Cat# 109-006-129; Jackson ImmunoResearch Labs) for 5 days in humidified incubator with 5% CO_2_. Proliferation was assessed by the MTT-based cell proliferation (Cat# G3580; Promega Corporation) kit as mentioned above and supernatant was also collected. IgG and IgM antibody secretion from the supernatant of stimulated cells was measured by ELISA using purified anti-human IgG and IgM (Cat# 109-005-006 and Cat# 109005-043) and peroxidase-conjugated anti-human IgG and IgM (Cat# 109-035-006 and Cat# 109-035-043) antibody pairs from Jackson ImmunoResearch Labs.

### Histological evaluation of biopsy of xenograft and explanted xenograft

Paraffin sections from multiple biopsies and sections of explanted xenografts were stained with haematoxylin and eosin for light microscopy. Sections were analysed semi-quantitatively for the presence of haemorrhage, necrosis, thrombosis and cellular infiltrates. Frozen sections of donor pig heart biopsy were immunostained with mouse anti-CD141 (clone PBS-0; 1:2,000, Abcam, Cambridge, MA, USA) or mouse IgG1 antibody (1:200, Abcam). Detection of CD141 was done using the EnVision System–HRP-Rabbit antibody (Cat# K4008; Dako, Carpinteria, CA, USA) followed by DAB+ Chromogen.

### Data analysis

The data here report individual results from each animal, therefore no aggregate statistical tests or randomization was used. The sample size was limited to five animals because this number of experiments turned out to be sufficient for evaluating the general potential of novel transplantation protocols in previous studies without overdoing costly and ethically critical experiments involving non-human primates. These studies were not blinded and all animals were of same genetic profile (except the promoter of *TBM* gene in donor pigs was ICAM in baboons #110, #210 and #910 and *TBM* in baboons #510 and #15009), and received same immunosuppressive regimen. GraphPad Prism or Microsoft Excel was used to generate all the graphs.

## Additional information

**How to cite this article:** Mohiuddin, M. M. *et al*. Chimeric 2C10R4 anti-CD40 antibody therapy is critical for long-term survival of *GTKO.hCD46.*hTBM** pig-to-primate cardiac xenograft. *Nat. Commun.* 7:11138 doi: 10.1038/ncomms11138 (2016).

## Figures and Tables

**Table 1:** Immunosuppressive regimen.

Agent	Dose	Timing	Route	Pre-treatment	Purpose
**Induction**					
Anti-CD20	19 mg kg^−1^	Days −7, 0, 7 and 14	i.v. infusion	Solu-Medrol, Benadryl, H2 blocker	To deplete B cells
ATG	5 mg kg^−1^	Days −2, and −1	i.v. infusion	Solu-Medrol, Benadryl, H2 blocker	To reduce number of T cells
Anti-CD40 (clone 2C10R4)	50 mg kg^−1^ for 100 days–1 year, then slowly tapered off		Slow i.v. infusion	None	Co-stimulation blockade. Suppression of both B- and T-cell response
CVF	50–100 U kg^−1^	Days −1, 0 and 1	i.v.	None	To inhibit complement activity
					
**Maintenance**					
Anti-CD40 (clone 2C10R4)	10–50 mg kg^−1^*	Weekly	Slow i.v. infusion	None	Co-stimulation blockade. Suppression of both B- and T-cell response
MMF	20 mg kg per 2 h	BID, daily	i.v. infusion	None	BID daily
Solu-Medrol	2 mg kg^−1^	BID tapered off in 7 weeks	i.v.	None	Suppress inflammation
Aspirin	81 mg		Oral	None	Prevent platelet aggregation
Heparin	50–400 U h^−1^	Continuous	i.v. infusion	None	Maintain ACT 2 × normal and prevent inflammation
					
**Supportive**					
Ganciclovir	5 mg kg^−1^ per day	Daily	i.v. infusion		For CMV prophylaxis
Cefazolin	250 mg	Daily for 7 days and whenever needed	i.v.	None	Antibiotic cover
Epogen	200 U kg^−1^	Day −7 to 7 then weekly	i.m. or i.v.	None	To increase haematocrit

BID, twice daily; CMV, Cytomegalovirus; CVF, cobra venom factor; i.m., intramuscular; i.v., intravenous.

^*^Anti-CD40 antibody dose was reduced either from 50 to 25 mg kg^−1^ on day 100 (*n*=2) or completely tapered off starting from day 365 (*n*=2).
